# Hybrid Feature Fusion-Based High-Sensitivity Fire Detection and Early Warning for Intelligent Building Systems

**DOI:** 10.3390/s23020859

**Published:** 2023-01-11

**Authors:** Shengyuan Xiao, Shuo Wang, Liang Ge, Hengxiang Weng, Xin Fang, Zhenming Peng, Wen Zeng

**Affiliations:** 1 School of Information and Communication Engineering, University of Electronic Science and Technology of China, Chengdu 611731, China; 2 School of Chemistry and Chemical Engineering, Southwest Petroleum University, Chengdu 610500, China; 3 School of Mechanical and Electronic Engineering, Southwest Petroleum University, Chengdu 610500, China; 4 School of Materials Science and Engineering, Chongqing University, Chongqing 400044, China

**Keywords:** early fire warning, hybrid feature fusion, intelligent building system, D-S evidence theory

## Abstract

High-sensitivity early fire detection is an essential prerequisite to intelligent building safety. However, due to the small changes and erratic fluctuations in environmental parameters in the initial combustion phase, it is always a challenging task. To address this challenge, this paper proposes a hybrid feature fusion-based high-sensitivity early fire detection and warning method for in-building environments. More specifically, the temperature, smoke concentration, and carbon monoxide concentration were first selected as the main distinguishing attributes to indicate an in-building fire. Secondly, the propagation neural network (BPNN) and the least squares support vector machine (LSSVM) were employed to achieve the hybrid feature fusion. In addition, the genetic algorithm (GA) and particle swarm optimization (PSO) were also introduced to optimize the BPNN and the LSSVM, respectively. After that, the outputs of the GA-BPNN and the PSO-LSSVM were fused to make a final decision by means of the D-S evidence theory, achieving a highly sensitive and reliable early fire detection and warning system. Finally, an early fire warning system was developed, and the experimental results show that the proposed method can effectively detect an early fire with an accuracy of more than 96% for different types and regions of fire, including polyurethane foam fire, alcohol fire, beech wood smolder, and cotton woven fabric smolder.

## 1. Introduction

Over the past few years, building fires have been one of the most common and frequent types of fire, causing severe property losses and fatalities [1,2,3]. Thus, it has become extremely important and imperative to effectively decrease and control the risk of fire in buildings. One of the most critical technologies is early fire warning, and both academia and industry are exploring approaches that enable the prompt detection and accurate determination of scene conditions in the early stages of building fires [4]. Furthermore, with the development of intelligent buildings, an effective early fire detection and warning system is also an essential prerequisite to provide safe, healthy, and comfortable residential or working conditions.

For achieving early fire detection and warning inside building environments, many algorithms have been presented, which can be mainly divided into three categories, namely optical, smoke, and carbon-monoxide sensors. Li et al. [5,6] conducted a fire detection algorithm performance evaluation based on image complexity, which could accurately determine the detection level of the detection algorithm under different image complexity conditions. Li et al. [7] proposed a new hybrid model for fire prediction based on the visual information of RGB images. The model achieves promising performance, which also shows the potential to monitor the constant changes in a building fire through the continuous processing of images of flames. Xie et al. [8] employed an early fire detection method for shielded indoor environments based on fire light reflection characteristics and established a multi-expert system by extracting the change characteristics of the fire area with light reflection. They then verified the method based on a large data evaluation system. However, in the above methods, they ignored the early stages of the fire that may not involve the open flame but cannot determine the correct scene conditions in time. The temperature, another major feature of fire, has also attracted significant attention. Ji et al. [9] proposed a machine-learning-based real-time prediction method for monitoring physical parameters for early fire warnings based on temperatures. The results show that the trained intelligences improve the accuracy and reliability of building fire warnings. Sun et al. [10] verified a bio-inspired artificial intelligence algorithm driven by temperature data to detect fire in 3D spaces. Yusuf et al. [3] presented a linearly regressive artificial-neural-network-based technique to predict temperature increases caused by building fire environments. This method predicts temperature ranges in a burning compartment based on the historic fire behavior data modelled via a neural network algorithm. Garrity et al. [11] exploited a compact embedded artificial neural network (ANN) with a second-stage classifier, reading temperature data from the in-built thermocouple, to produce the output of predicted temperatures. However, this technical approach, namely its ability to judge temperature fluctuations in special environments, still requires further improvement. To compensate for the deficiencies of the feature parameter, Zhang et al. [12] proposed DBN-R-LSTM-NN to classify and predict fire smoke and other features based on data collected from IoT sensors. Pincoot et al. [13] designed a vision-based indoor fire and smoke detection system with small training and test datasets, whereby different pixel density images, high-density smoke environments, and flame density scenes were recognized. Qiu et al. [14] developed a distributed feedback carbon monoxide (CO) sensor based on lasers used for the early warning detection of fire and verified the reliability of this sensor via different experiments. Li et al. [15] studied and proposed an early fire detection method based on a gas turbulent diffusion (GTD) model and particle swarm optimization (PSO). The experimental results verify that the sensor system has good fire detection and location performance. Chen et al. [16] proposed a fast and cost-effective indoor fire alarm system, which is used to find the fire, carbon monoxide, smoke, temperature, and humidity data in real time and conduct effective data analysis and classification. For smoke detection, the influence of smoke and dust in the environment represents a challenge.

Although vision-based sensing systems have shown good performance, massive and complex datasets often delay the time in the decision-making process [17]. Thus, visual-based sensing systems are not appropriate for early fire detection where decision making needs to be conducted in limited time. Additionally, vision-based sensing systems are inappropriate inside buildings because of the size and cost of devices, as well as privacy concerns that make them unsuitable for installation in multiple rooms and private homes. Meanwhile, single chemical sensor fire detectors are susceptible to interference from environmental conditions such as electromagnetic waves, water vapor, dust, cigarette smoke, and cooking smoke [18], which means that most smoke detectors will have false alarms when they detect these interfering conditions. Similarly, single temperature sensor devices still suffer from the misjudgment of environmental temperature fluctuations. They cannot effectively distinguish between early fire signals and environmental interference signals and cannot properly send early fire warning signals.

Due to the rapid development of machine learning, many intelligent algorithms have been presented to fuse several fire feature parameters [19,20,21,22]. This method overcomes the singularity and instability of the traditional threshold judgment method, which can significantly improve the accuracy of fire detection. Therefore, this paper proposes a hybrid feature fusion-based early fire detection method. First, by means of the distributed wireless sensing network, a hybrid feature collection system is developed to collect the smoke concentration, temperature, and CO concentration. On this account, a hybrid feature fusion algorithm, combining an error back propagation (BP) neural network with a least square support vector machine (LSSVM), is proposed. Then, the genetic algorithm (GA) and particle swarm optimization (PSO) are applied to obtain the optimal parameters in the data fusion process of the two algorithms, in order to achieve the optimized feature-level fusion results. Furthermore, the two algorithms are treated as subevidence bodies and the output results are adopted as the basic probability assignment of the D-S evidence theory for decision-level fusion to realize highly sensitive and reliable early fire warnings.

## 2. Suggestions and Methodology

**Figure 1:**
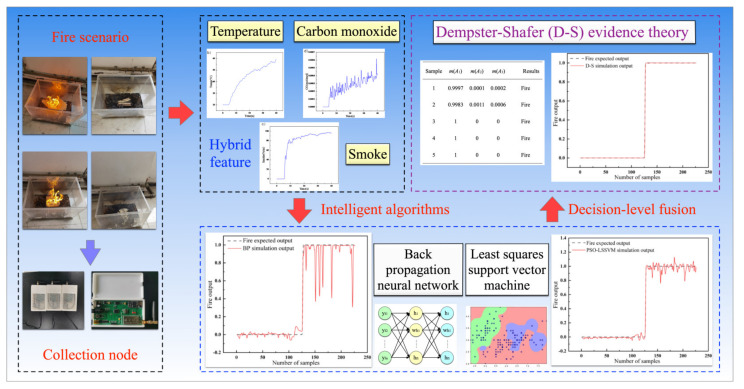
Overall design of the early fire detection and warning system.

The process of the hybrid feature data fusion algorithm is shown in Figure 1. The smoke concentration, temperature, and carbon monoxide concentration are obtained after collecting nodes and they are then transmitted to the data processing center via LoRa as inputs of the genetic algorithm–back propagation neural network (GA-BPNN) and the particle swarm optimization–least squares support vector machine (PSO-LSSVM). The two models both obtain three outputs: fire, smolder, and no fire. Then, the results of the feature layer fusion are integrated by the D-S evidence theory to output the final decision.

### 2.1. Genetic Algorithm–Back Propagation Neural Network Fire Warning Model

**Figure 2:**
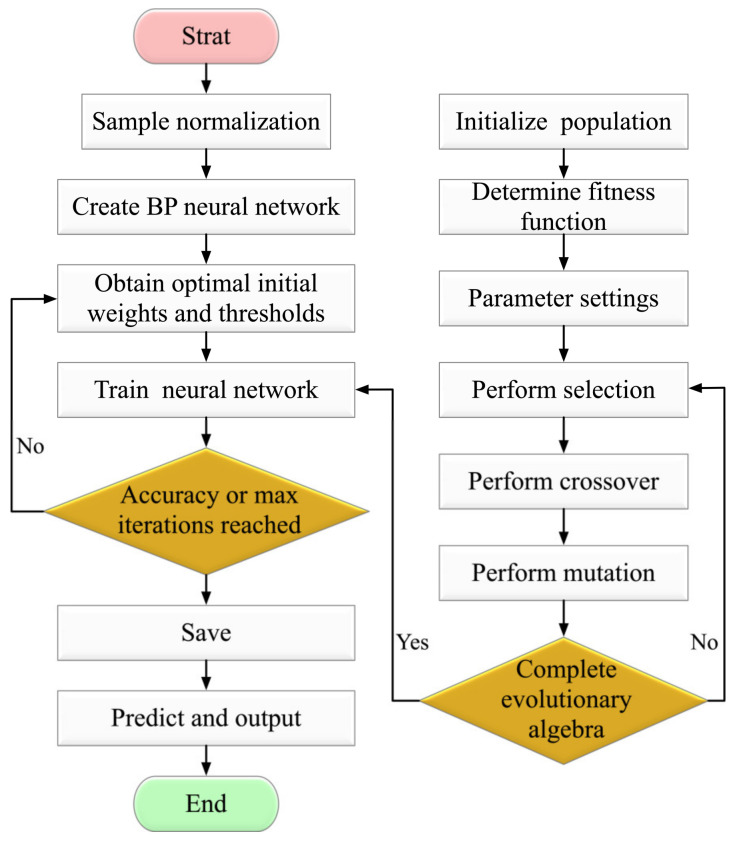
The BPNN process with GA optimization.

The BPNN algorithm optimized by GA is a continuous and iterative process used for searching the best weight and threshold [19]. The process of the BPNN algorithm optimized by GA is shown in Figure 2.

The BPNN is a multi-layer forward feedback network for error correction, which includes the forward propagation of the signal and backward propagation of the error. The error excitation function is a sigmoid function, as shown in Equation (1).

(1) $$\begin{array}{l} Y_{i}=\frac{1}{1+\exp\left[-\left(\sum_{i=1}^{n}w_{ij}x_{i}+w_{j0}\right)\right]}\\ Y_{k}=\sum_{k=1}^{N}w_{kj}Y_{j}+w_{k0} \end{array}$$

where *Y_k_* is the *k*th variable of the output layer, *Y_j_* is the *j*th variable of the hidden layer, *x_i_* is the *i*th input variable, *N* is the number of output neurons, *n* is the number of hidden neurons, *w*_kj_ is the weight between the output layer and the hidden layer, and *w*_ji_ is the weight between the input layer and the hidden layer.

The gradient descent method is often used to optimize the parameters of neural networks. However, BPNN is difficult to converge and may fall into local extremes. Since the GA [23] can reduce the solution space of the neural network, the global optimal solution can be obtained more quickly and stably by the GA-BPNN [24]. The GA uses selection, crossover, and mutation to generate several initial populations of defined encoding lengths in a random number [25].

In the GA optimization strategy, the best realized individuals of the new generation will be considered as a result of the execution and GA setups shown in Table 1. The optimal parameters obtained by GA are then brought into the BPNN for training.

### 2.2. Particle Swarm Optimization–Least Squares Support Vector Machine Fire Warning Model

**Figure 3:**
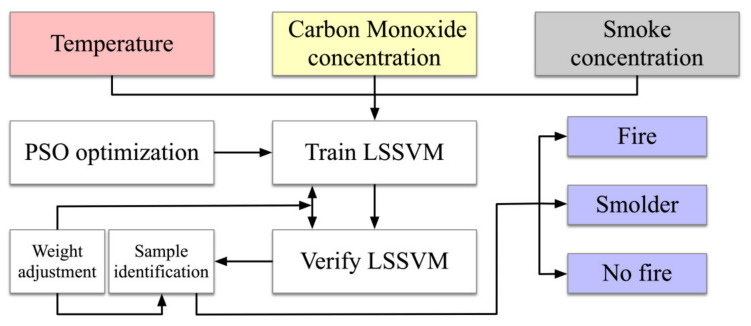
The PSO-LSSVM fire warning algorithm model.

The inputs to the LSSVM are the smoke concentration, temperature, and carbon monoxide concentration. The penalty factor *C* and the kernel parameter *σ* of the LSSVM are optimized by PSO. The simulation outputs are compared with the expected outputs to verify the feasibility of the algorithm, and the design is shown in Figure 3.

In support vector machines (SVMs), the dot product algorithm in the high-level feature is replaced by the kernel function [26].

SuykensJ and Vandewalle [27] further proposed the least squares support vector machine (LSSVM) for solving pattern classification and regression prediction problems [28]. A nonlinear function *φ*(*x_i_*) is used to map the input to the feature space, and the nonlinear function estimation modeling is shown in Equation (2).

(2) $$f\left(x\right)=b+\left(\emptyset \left(x\right),w\right)$$

where *w* is the weight vector and *b* is the bias term.

The evaluation problem is described as the optimization problem based on the principle of structured risk minimization and the Lagrange function is constructed, as shown in Equation (3), to solve the optimization problem.

(3) $$L_{LSSVM}=\frac{1}{2}w^{2}+\frac{1}{2}\gamma \overset{N}{\underset{i=1}{\sum }}e_{i}^{2}-\overset{N}{\underset{i=1}{\sum }}\alpha_{i}\left[\left(w,\emptyset \left(x_{i}\right)\right)+b+e_{i}-y_{i}\right]$$

where *a_i_* is the Lagrange multiplier.

The linear problems can be simplified by eliminating *w* and *e_i_*, as described in Equation (4).

(4) $$\left[\begin{array}{cc} 0 & E^{T}\\ E & Ώ+\frac{1}{\gamma }E \end{array}\right]\left[\begin{array}{c} b\\ a \end{array}\right]=\left[\begin{array}{c} 0\\ y \end{array}\right]$$

where *y* = [*y*_1_, …, *y*_N_]*^T^*, *a* = [*a*_1_, …, *a*_N_]*^T^*, *E* = [1, …, 1]*^T^*, and *Ώ* is a symmetric matrix equation for the *N* × *N* as shown in Equation (5).

(5) $$\Omega_{ij}=K(x_{i},x_{j})=\emptyset {\left(x_{i}\right)}^{T}\emptyset (x_{j})i,j=1,2,\ldots ,N$$

where *K*(*x_i_*, *y_j_*) is the kernel function that satisfies the Meser condition [29].

Finally, the LSSVM can be described, as shown in Equation (6).

(6) $$y\left(x\right)=\left(w,\emptyset \left(x\right)\right)+b=\overset{n}{\underset{i=1}{\sum }}\alpha_{i}\emptyset \left(x_{i}\right)\cdot \emptyset \left(x\right)+b=\overset{n}{\underset{i=1}{\sum }}\alpha_{i}K\left(x_{i},x\right)+b$$

The four main forms of the LSSVM kernel function are shown in Table 2.

The PSO algorithm simulates a bird in a flock by designing a massless particle with position and velocity attributes [30]. The individual extremes are matched with the current global optimal solution shared by other particles in the global to adjust the speed and position [31].

The PSO will initialize to a group of random particles and find the optimal solution by iteration [21]. The particle update is tracked (*p_best_*, *g_best_*) in iteration by Equations (7) and (8).

(7) $$v_{i}=v_{i}+c_{1}\times rand()\times \left(pbest_{i}-x_{i}\right)+c_{2}\times rand()\times \left(gbest_{i}-x_{i}\right)$$

(8) $$x_{i}=x_{i}+v_{i}$$

where *i* = 1, 2, …, *N*, and *N* is the total number of particles. *V_i_* is the speed of the particle. Rand ( ) is a random number between 0 and 1. *X_i_* is the current position of the particle. *C*_1_ and *c*_2_ are the learning factors of *c*_1_ = *c*_2_ = 2. The maximum value of *v_i_* is *Vmax* (>0); if *v_i_* > *Vmax*, then *v_i_* = *Vmax*.

**Figure 4:**
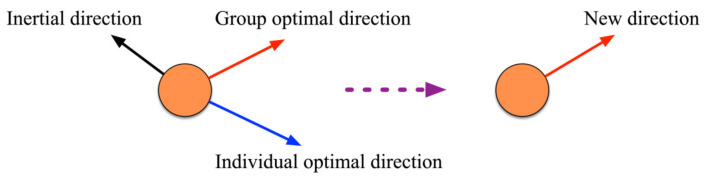
Schematic diagram of particle search for optimization.

According to the update of the velocity *v*, *c*_1_ is aimed towards the local optimal solution and *c*_2_ is aimed towards the global optimal solution. The representation is shown in Figure 4.

**Figure 5:**
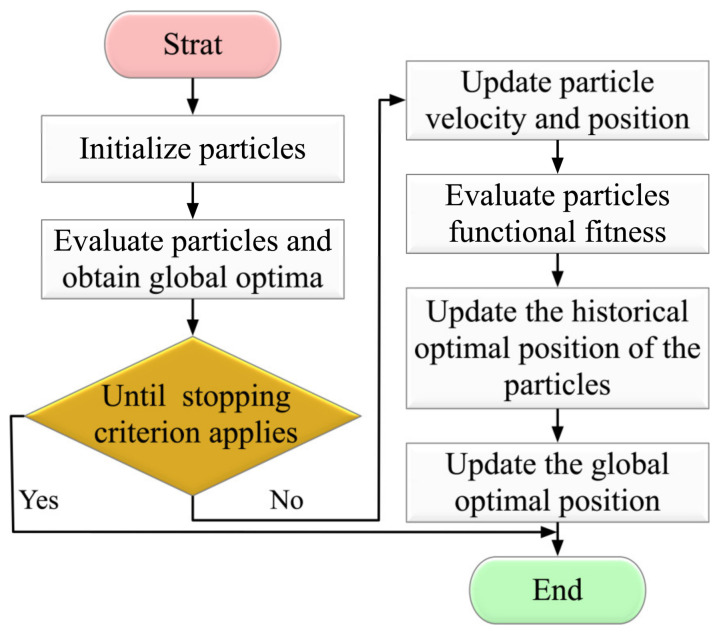
Particle swarm optimization process.

The design steps of the PSO are shown in Figure 5.

The initialization of the PSO algorithm is shown in Table 3. The fire detection uses the smoke concentration, temperature, and carbon monoxide concentration as inputs for training and testing samples. Then, the samples are trained by the LSSVM. The optimal penalty factor *c* and the optimal kernel function width factor *σ* of the LSSVM are obtained by the PSO optimization search. The partial fusion results of another group of samples can be obtained as the original fusion results for adjustment.

To assess the performance of the BPNN, the GA-BPNN, and the PSO-LSVM model, three evaluation indicators, i.e., the mean square error (MSE), the root mean square error (RMSE), and the mean absolute error (MAE), are adopted [20]. The MSE, RMSE, and MAE are calculated, as shown in Equations (9)–(11). The smaller the MSE, RMSE, and MAE, the smaller the error.

(9) $$RSE=\frac{1}{N}\sum_{m=1}^{N_{d}}{\left({\hat{y}}_{m}-y_{m}\right)}^{2}$$

(10) $$RMSE=\sqrt{\frac{1}{N_{d}}\sum_{m=1}^{N_{d}}{\left({\hat{y}}_{m}-y_{m}\right)}^{2}}$$

(11) $$MAE=\frac{1}{N_{d}}\sum_{m=1}^{N_{d}}\left|\left({\hat{y}}_{m}-y_{m}\right)\right|$$

where *N_d_* is the amount of data, $y_{m}$ is the actual value of data, and ${\hat{y}}_{m}$ is the simulation output of the model.

### 2.3. Decision-Level Feature Data Fusion

The D-S evidence theory [32,33] is specialized in solving uncertainty problems [34,35]. It fuses confidence functions obtained by different algorithms to make decisions using the combination rules of the evidence theory. The system will make a final decision based on new evidence according to the decision rules.

Suppose *U* is an identification frame, the function *m*: 2^Θ^ → [0, 1] satisfies Equation (12).

(12) $$m(\phi )=0,\sum_{A\subseteq \Theta }m(A)=1$$

*m* is the basic probability assignment function based on 2^Θ^ and *m*(*A*) is the basic probability assignment of proposition *A*, indicating the degree of confidence in the probability assignment.

The confidence function indicates the total degree to which the obtained evidence supports the information grading. The function *Bal* is defined by ∀*A* ⊆ Θ and $Bal\left(A\right)=\sum_{B\subseteq A}m\left(B\right)$. 2^Θ^ → [0, 1] is the reliability function on Θ. For ∀*A* ⊆ Θ if *m*(*A*) > 0, *A* is the focal element of the reliability function *Bel* and the union of all the focal elements in the frame Θ is the kernel.

The likelihood function describes the degree to which the obtained evidence cannot reject the score. A *pl*: 2^Θ^ → [0, 1] is defined in Equation (13):

(13) $$pl(A)=1-Bel(A)=\sum_{B\cap A\neq \phi }M(B)$$

The confidence functions of *Bel*1 and *Bel*2 are demonstrated. *M*_1_ and *m*_2_ are the corresponding basic probability assignment functions. The focal elements are *A*_1_, *A*_2_, …, *A*_n_ and *B*_1_, *B*_2_, …, *B*_n_. The new probability *M* can be obtained according to the D-S evidence theory shown in Equation (14):

(14) $$M=\left\{\begin{array}{l} 0,C=\phi \\ \frac{1}{1-K}\sum_{A_{i}\cap B_{j}=C}m_{1}(A_{i})m_{2}(B_{j}),C\neq \Phi \end{array}\right.$$

The calculation of *K* is shown in Equation (15).

(15) $$K=\sum_{A\cap B=\phi }m_{1}(A)m_{2}(B)$$

On the one hand, if *K* ≠ 1, the two are compatible, and basic probability assignment can be conducted. On the other hand, if *K* = 1, then *m*_1_ and *m*_2_ are contradictory or cannot combine.

The decision of the D-S evidence theory followed the rules shown below, assuming that *A*_1_ ⊂ Θ, *A*_2_ ⊂ Θ satisfies Equations (16) and (17).

(16) $$m(A_{1})=\max\left[m\left(A_{i}\right),A\subset \Theta \right]$$

(17) $$m(A_{2})=\max\left[m\left(A_{i}\right),A_{i}\subset \Theta \right],A_{i}\neq A_{1}$$

If Equations (18) and (20) are satisfied as below:

(18) $$m(A_{1})-m(A_{2})>\varepsilon_{1}$$

(19) $$m(\Theta )<\varepsilon_{2}$$

(20) $$m(A_{1})>m(\Theta )$$

where *ε*_1_ and *ε*_2_ are the preset thresholds, then *A*_1_ is the determined result by the D-S evidence theory.

The training error *e* of the GA-BPNN and the PSO-LSSVM is calculated as part of the basic probability assignment shown in Equation (21).

(21) $$e=\frac{1}{2}\sum {\left(t_{i}-y_{i}\right)}^{2}$$

where *t*_i_ and *y*_i_ are the expected and simulated outputs in the GA-BP and the PSO-LSSVM.

The identification framework of the evidence theory Θ = {*A*_1_, *A*_2_, *A*_3_}, *A*_1_, *A*_2_, *A*_3_ represents the fire, smolder, and no fire. In the portfolio of evidence *E* = {*E*_1_, *E*_2_}, *E*_1_ and *E*_2_ denote the outputs of the GA-BP and the PSO-LSSVM, respectively. *m*_1_(*A*_1_), *m*_1_(*A*_2_), and *m*_1_(*A*_3_) represent the basic probability assignments of the GA-BP for the three outputs of fire, smolder, and no fire, respectively. *m*_2_(*A*_1_), *m*_2_(*A*_2_), and *m*_2_(*A*_3_) represent the basic probability assignments of the PSO-LSSVM for the three outputs of fire, smolder, and no fire, respectively.

The final basic probability assignment of each element obtained from Equation (21) is shown in Equation (22) below:

(22) $$m_{j}(A_{i})=\frac{y(A_{i})}{\sum_{i=1}^{3}y(A_{i})+e}(j=1,2)$$

Then, the D-S evidence theory fusion rule is described in Equation (23).

(23) $$m(A_{z})=m_{1}(A_{i})⊕m_{2}(A_{j})=\left\{\begin{array}{l} \frac{1}{1-K}\sum_{A_{i}\cap A_{j}=A_{z}}m_{1}(A_{i})m_{2}(A_{j}),\forall A_{Z}\subseteq \Theta ,A_{z}\neq \phi \\ 0,A_{Z}=\phi \end{array}\right.$$

Equation (24) is then used to calculate *K*:

(24) $$K=\sum_{A_{i}\cap A_{j}=\phi }m_{1}(A_{i})m_{2}(A_{j})$$

## 3. Results and Discussions

### 3.1. Experimental Setup

**Figure 6:**
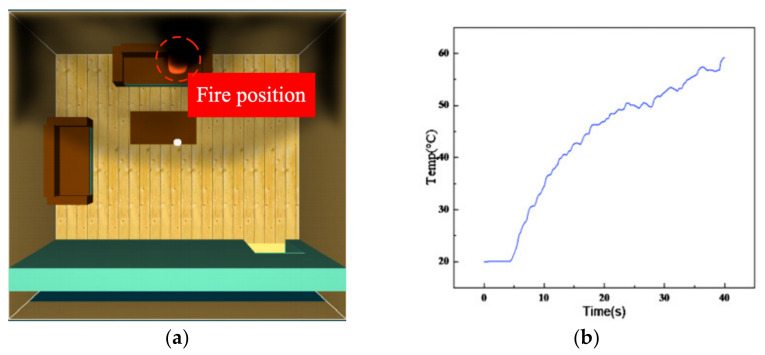

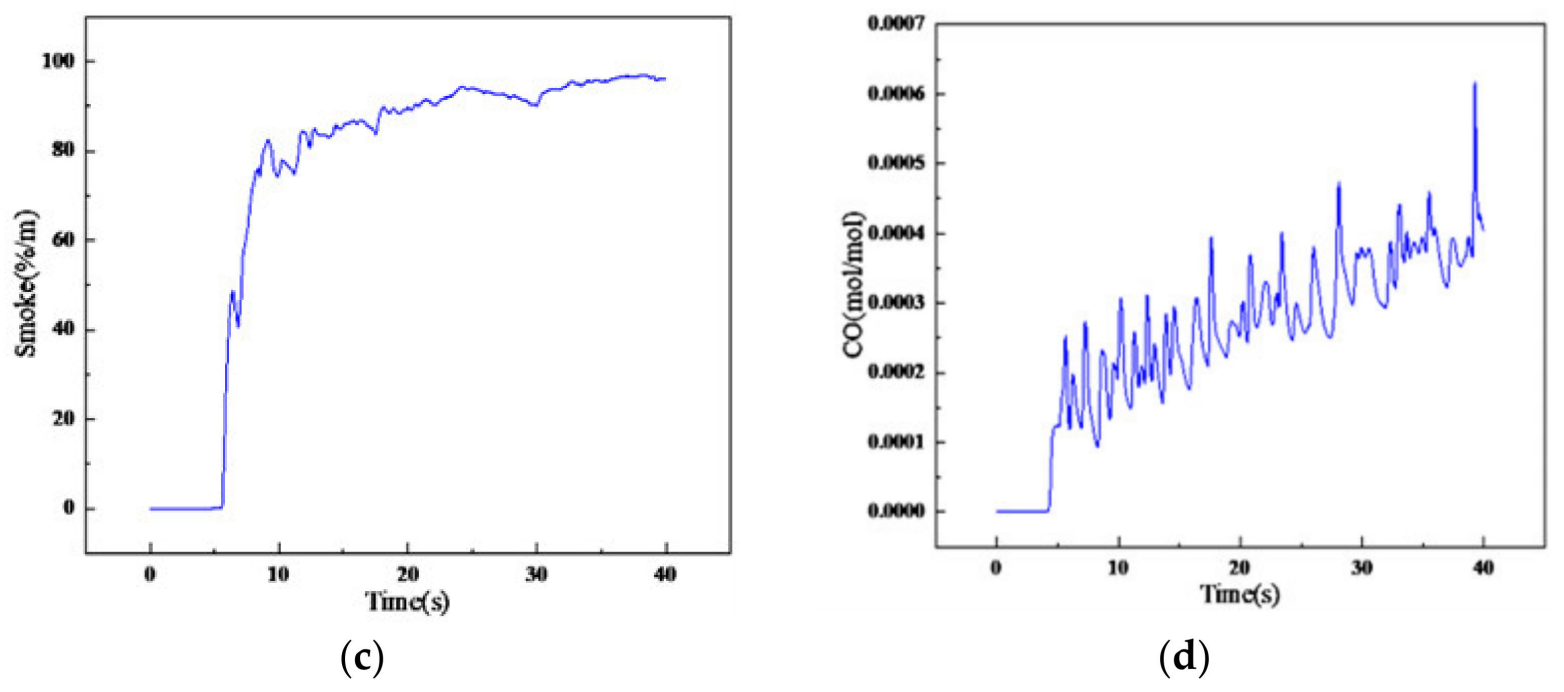
The simulation analysis of in-building fire features: (**a**) fire model; (**b**) temperature change; (**c**) smoke concentration change; and (**d**) carbon monoxide concentration.

Firstly, the fire dynamics simulator (FDS) [36,37] was employed to simulate in-building fires, as shown in Figure 6a. The large eddy simulation (LES) in the FDS was used by the Smagorinsky subgrid model to solve the Navier–Stokes equations for the turbulence with a low Mach number caused by the fire phenomenon. The simulated setting was a no-wind scenario. In this figure, the burning duration was set to 40 s, and the combustion products in the space are presented in Figure 6b–d. We can see that the temperature, smoke concentration, and CO concentration changed slowly in the first five seconds, and then they began to rise significantly. At the end of the 40 s burn time, the temperature achieved the maximum, while the smoke variation stabilized, and the CO concentration continued to increase. Therefore, according to the results of the building fire simulation, these three feature parameters were selected as the monitoring parameters of the fire warning system.

**Figure 7:**
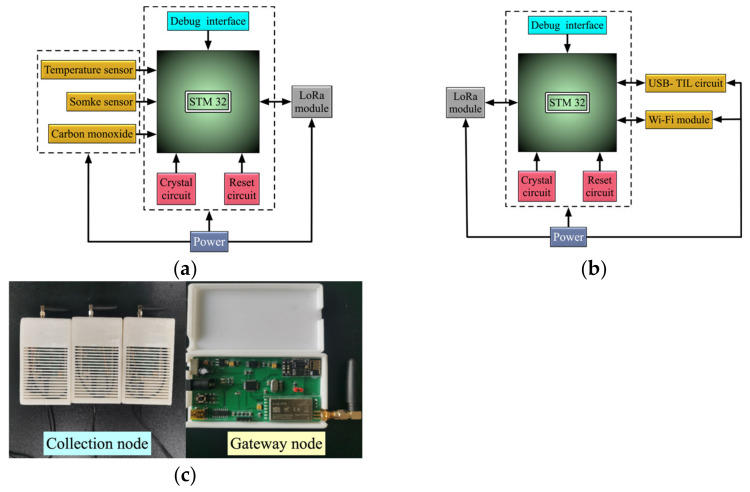
The fire detection and warning system: (**a**) collection node design; (**b**) gateway node design; (**c**) hardware.

Three key parameters, i.e., temperature, smoke concentration, and carbon monoxide concentration, were extracted from the experimental data to form the basic datasets. Then, we divided the outputs of the hybrid feature fusion model into three classes (open fire, smolder, and no fire). On this basis, (1,0,0), (0,1,0), and (0,0,1) were modeled as the ideal representations of open fire, smolder, and no fire, respectively. Subsequently, we developed a combustion product collection system, including the combustion product collection node and gateway node, as shown in Figure 7, to obtain the real experimental data generated by small simulated in-building fires, where 800 sets of data were collected. The feature data in the datasets were derived from combustion products of different materials, while each dataset included the temperature (Temp), smoke concentration (Smoke), and carbon monoxide concentration (CO). After that, these data were further divided into 600 sets of training data and 200 groups of test data. The training data were utilized to train the model and the optimal network training model could be obtained after consecutive iterations with learning rates of 0.001. The test data were employed to demonstrate the effectiveness.

### 3.2. Results of Open Fire

**Figure 8:**
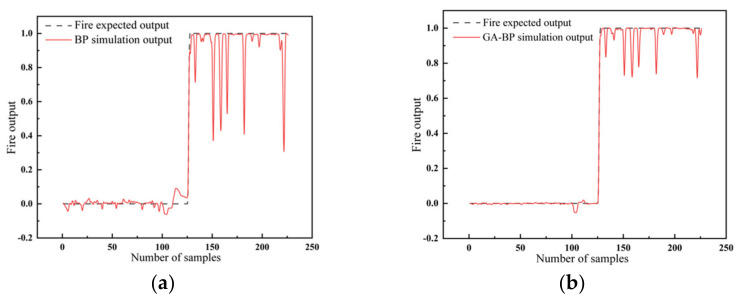
The output results of fire with (**a**) the BPNN and (**b**) the GA-BPNN.

The fire test data were substituted into the already trained BPNN, and the results are shown in Figure 8a. The comparison reveals that the simulation output of the BPNN differed significantly from the expected output of fire. However, the network model optimized by the GA could obtain the optimal weights and thresholds, which we inputted into the BPNN for training. The outputs are shown in Figure 8b. It can be seen that the difference between the simulated and expected outputs was reduced.

**Figure 9:**
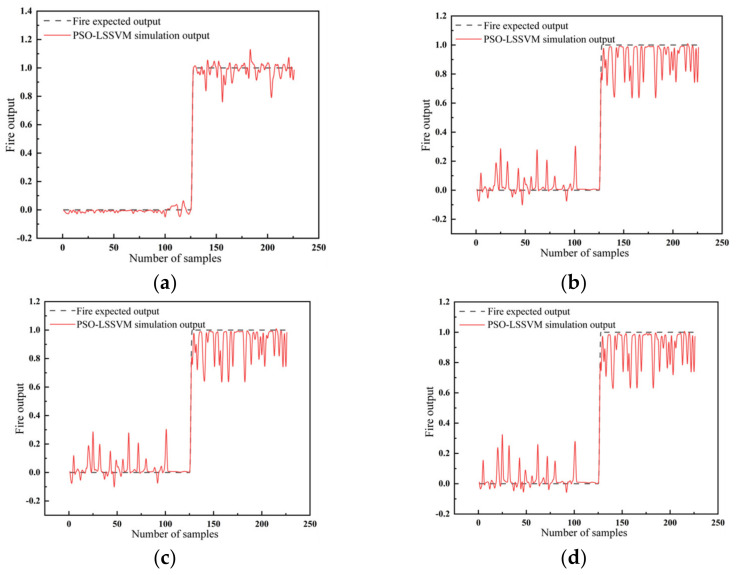
The output results of fire with the LSSVM for different kernel functions: (**a**) the radial basis kernel function; (**b**) the sigmoid kernel function; (**c**) the polypolynomial kernel function; (**d**) the linear kernel function.

The PSO-LSSVM fire warning model adopted four kernel functions: (1) the radial basis kernel function; (2) the sigmoid kernel function; (3) the polypolynomial kernel function; (4) and the linear kernel function. The simulation results of fire based on the four types of kernel functions were compared and analyzed with the expected outputs, as shown in Figure 9a–d. It is clear that the radial basis kernel function could perform better than the other three kernel functions. Thereby, it delivered the best data fusion effects.

### 3.3. Results of the Smolder

**Figure 10:**
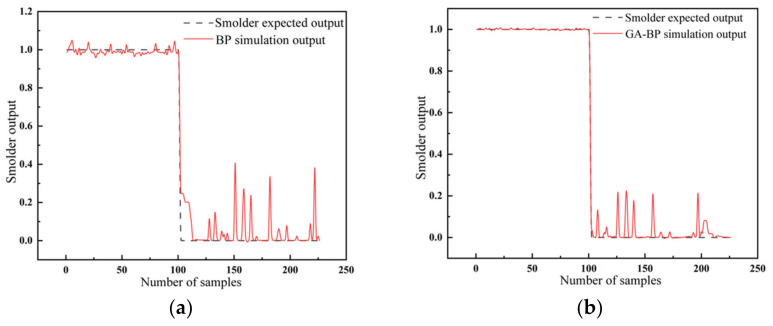
The output results of smolder with (**a**) the BPNN and (**b**) the GA-BPNN.

Figure 10 shows that the simulation results of the BPNN optimized by GA had better stability. Compared with the original model, the difference between the simulation results and the expected results was significantly reduced.

**Figure 11:**
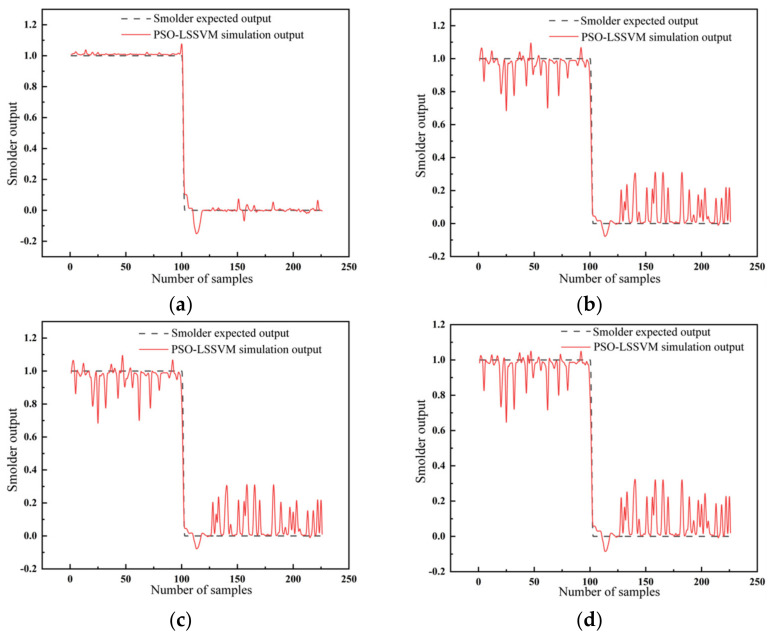
The output results of smolder with the LSSVM for different kernel functions: (**a**) radial basis kernel function; (**b**) sigmoid kernel function; (**c**) polypolynomial kernel function; (**d**) linear kernel function.

Figure 11 illustrates the different output results of smolder with the LSSVM. It can be seen that the radial basis kernel function also obtained the most stable output results, representing the highest reliability and the best data fusion effects.

### 3.4. Model Performance Analysis

Table 4 shows the error MSE, RMSE, and MAE of the BPNN and the GA-BPNN to quantify the model performance. Table 4 also shows that the GA-BPNN had the smallest MSE, RMSE, and MAE, which indicates that the simulation output of the GA-BPNN was more suited to the expected output. The GA-BPNN also performed better than the original BPNN. Therefore, the GA-BPNN could also fuse the fire feature data in the feature layer.

Table 5 illustrates the calculation analysis of the MSE, RMSE, and MAE for different kernel functions. As shown in Table 4 and Table 5, the algorithm based on the RBF had the smallest MSE, RMSE, and MAE, indicating that the simulation output was closer to the expected output and the model performance was better than the others. Therefore, the PSO-LSSVM model based on the RBF was selected to fuse the feature layer data for the fire feature.

### 3.5. Results of the Proposed Method

According to the empirical definition *ε*_1_ = 0.5, the simulation outputs of five groups of test sample data were selected for comparison and analysis with the expected outputs using Equation (24). Table 6 shows three uncertainty data outputs from the GA-BPNN and one uncertainty output from the PSO-LSSVM. These results are ambiguous. So, these five groups of results were then assigned and calculated by the probability of the D-S evidence theory, and the results are shown in Table 7. The evidence indicates that the results of the fusion based on the D-S evidence theory were consistent with the expected outputs. Thus, the results of the D-S evidence theory fusing the two model treatments effectively addressed the ambiguity of the evidence and improved the reliability of the fused data.

**Figure 12:**
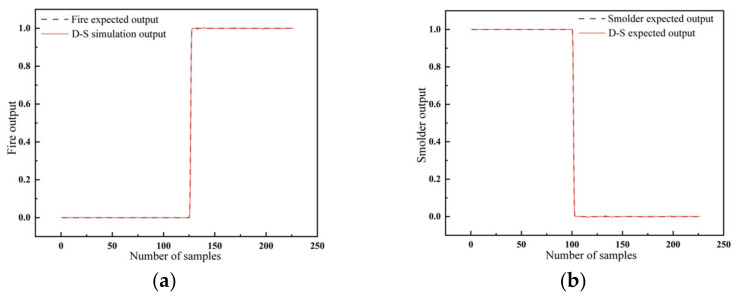
Comparative results of the D-S evidence theory fusion approach: (**a**) expected fire output and simulation outputs; (**b**) desired smolder output and simulation outputs.

A comparison of the simulated and expected outputs of the D-S evidence theory incorporating the GA-BP fire warning algorithm and the PSO-LSSVM fire warning algorithm is shown in Figure 12. The expected output and the fused output were basically the same. Additionally, the inferred results of the D-S evidence theory enabled the accurate determination of fire hazards. The combined prediction method, used to fuse the information from the above two networks after identification, provided results with higher accuracy than each single model.

## 4. Early Fire Warning Experiments

To verify the real-time performance of the presented early fire detection and warning system, we conducted four early fire warning experiments with different fire classes, i.e., polyurethane foam fire, alcohol fire, beech wood smolder, and cotton woven fabric smolder. A combustion experiment box was then placed inside the building and the fire detector was installed at 2.0 m above the combustion box.

### 4.1. Results of the Warning Time

**Figure 13:**
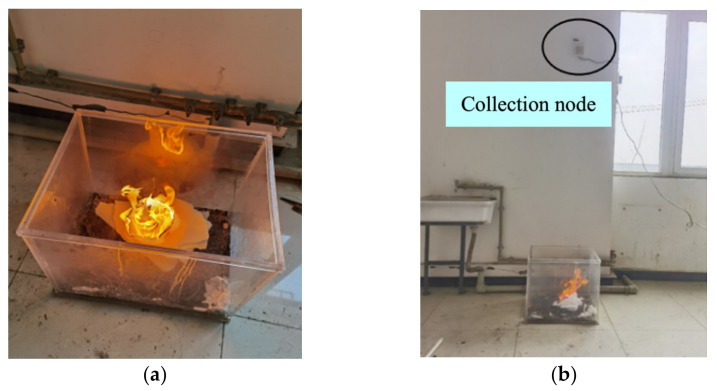
The polyurethane fire test: (**a**) the process of polyurethane combustion; (**b**) the fire scene.

Figure 13a shows the combustion process of polyurethane foam, and Figure 13b shows the fire scene. The polyurethane burned slowly at the beginning. However, a period later, the flame became larger, with a gradually increasing temperature and producing much smoke. The PC alarmed after 16 s.

**Figure 14:**
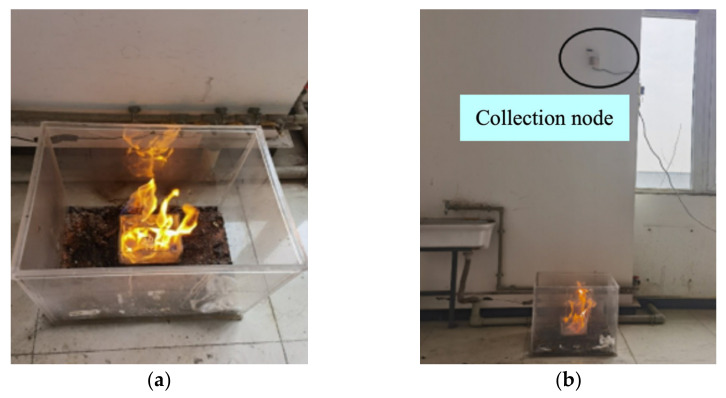
The alcohol fire test: (**a**) the process of alcohol combustion; (**b**) the fire scene.

Figure 14a shows the process of alcohol burns, and Figure 14b shows the scene of alcohol fire. The alcohol immediately burned when ignited. The flame became large and released a lot of heat, resulting in the temperature rising rapidly. However, there was no visible smoke. The PC alarmed after 18 s.

**Figure 15:**
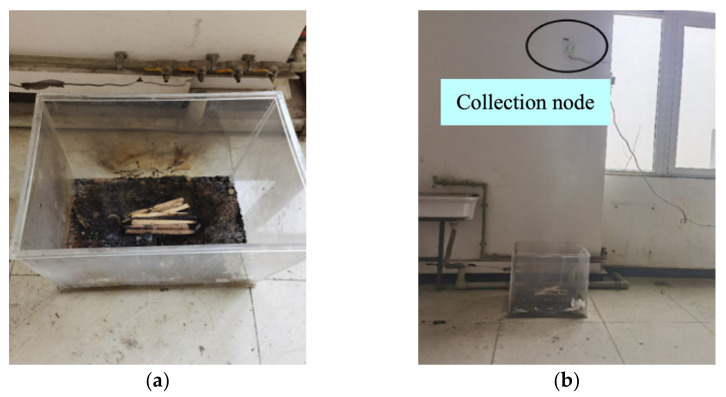
The beech wood smolder test: (**a**) the process of beech wood combustion; (**b**) the smolder scene.

Figure 15a shows the burn process of beech wood and Figure 15b shows the beech wood experiment with smolder. The smolder had no obvious fire but produced smoke particles with a slow rise in temperature. The PC alarmed after 22 s.

**Figure 16:**
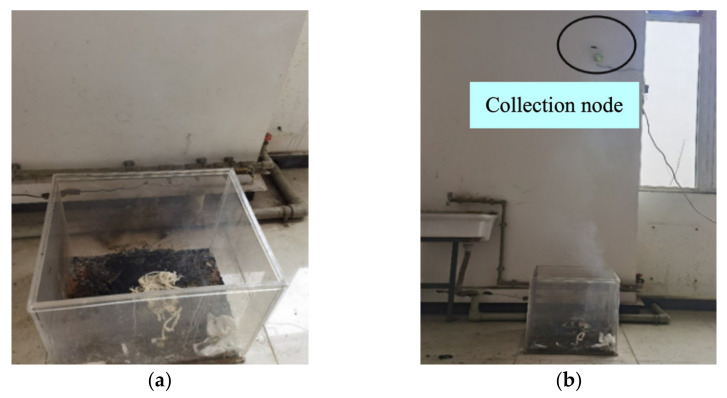
The cotton rope smolder test: (**a**) the process of cotton rope combustion; (**b**) the smolder scene.

Figure 16a shows the process when the cotton rope is ignited and smoldered, and Figure 16b shows the overall state of the cotton rope smolder. The smolder process produced a large amount of smoke, but the temperature did not rise significantly with a few sparks. The PC alarmed after 20 s.

### 4.2. Accuracy Verification of Alarm System

Fifty experiments were conducted on the above scenarios to verify the accuracy of the fire warning system. The data were collected using a single acquisition node for the features information generated from each burn experiment to verify whether the results in the PC match the real situation. A comparison between the realistic case and the fire warning system is shown in Table 8. The alarm accuracy of each type of experiment was shown to exceed 96%. The results show that the early fire warning model can ensure the detection and warning of different types of fire hazards.

### 4.3. Distributed Network Fire Response

**Figure 17:**
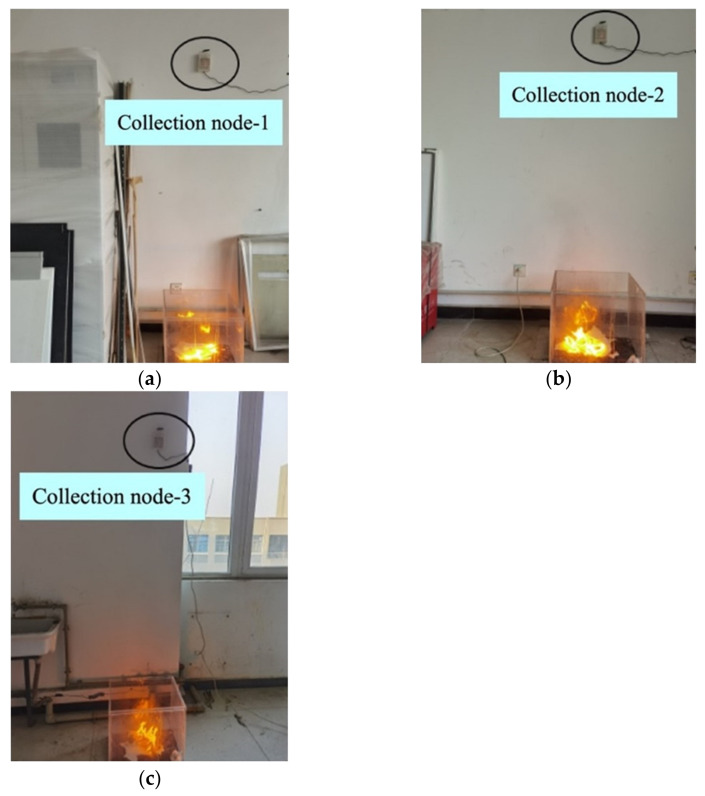
Distributed networking experiments: (**a**) Node 1; (**b**) Node 2; (**c**) Node 3.

The main purpose of the distributed network fire warning experiment is to verify whether the early fire warning system consisting of three data collection nodes can achieve the simultaneous detection of fires in multiple regions. Three fire feature data collection nodes were used to detect fires in three different regions (see Figure 17). Each collection node was connected to a gateway node via LoRa wireless spread spectrum technology, and the gateway node collected the environmental feature data acquired by each collection node. The fusion results of the GA-BP algorithm are shown in Table 9, the fusion results of the PSO-LSSVM algorithm are shown in Table 10, and the fusion results of the D-S evidence theory are shown in Table 11.

It is evident that the data obtained from different sensor nodes collected by the GA-BP and PSO-LSSVM algorithms were also ambiguous after calculation. However, the fusion of different results using the D-S evidence theory was shown to improve the reliability of the evidence.

## 5. Conclusions

This paper proposes a data fusion method with multiple features to achieve an in-building fire detection and early warning system. Firstly, the GA-BPNN and the PSO-LSSVM were employed to translate the smoke concentration, temperature, and CO concentration into probabilities of fire, smolder, and no fire. Subsequently, the feature data of the GA-BPNN and the PSO-LSSVM were fused by the D-S evidence theory to further improve the accuracy and the reliability of the fire warning algorithm. Finally, an early fire detection and warning system was developed. Small-scale in-building fire experiments have confirmed the effectiveness of the method, and the combined algorithm of the D-S evidence theory could significantly improve early fire detection. Additionally, the early fire warning system could accurately identify the fire signals from different types and regions. The early fire detection accuracy also exceeded 96%. However, the proposed method can only achieve fire recognition. It cannot be utilized to indicate the severity of fire. In addition, in this paper, limited kinds of experimental materials were employed for fire combustion. As a result, in future work, we plan to develop an end-to-end approach that can not only achieve fire detection but also predict the fire levels based on the featured data, and more kinds of experimental materials will be presented to demonstrate the effectiveness. Moreover, another valuable research interest is to identify the smoking behavior for avoiding false fire alarm by utilizing the hybrid feature fusion-based method.

## Figures and Tables

**Table 1:** The GA setups.

Types	Symbols	Interval
The coding length	*N*	20~200
The crossover probability	*P*_c_	0.4~0.99
The mutation probability	*\*	0.005~0.1
The number of terminated evolutionary generations	*\*	100~1000

**Table 2:** The forms of the kernel function.

Kernel Function	Forms
The polynomial kernel function	$K\left(x,x_{i}\right)={\left(x\cdot x_{i}+c\right)}^{N}$
The radial basis kernel function	$K\left(x,x_{i}\right)=\exp\left(\frac{\|x-x_{i}\|{}^{2}}{2\sigma^{2}}\right)$
The sigmoid kernel function	$K\left(x,x_{i}\right)=\tanh\left(\tau \cdot \left(x,x_{i}\right)+t\right),(\tau >0,t>0)$
The linear kernel function	$k\left(x,x_{i}\right)=x*x_{i}$

**Table 3:** The PSO algorithm initialization parameters.

Types	Symbols	Interval
The acceleration factor	*c*_1_	1.5
	*c*_2_	1.7
The maximum number of population evolution	*\*	300
The population size	*\*	30
The penalty factor range	*\*	0.1~1000
The kernel function width factor range	*σ*	0.01~1000

**Table 4:** The output error analysis.

Algorithm Model	Fire			Smolder		
	MSE	RMSE	MAE	MSE	RMSE	MAE
BP	0.0185	0.1361	0.0412	0.0083	0.0910	0.0362
GA-BP	0.0037	0.0612	0.0164	0.0025	0.0498	0.0140

**Table 5:** The output error analysis.

Kernel Function	Fire			Smolder		
	MSE	RMSE	MAE	MSE	RMSE	MAE
RBF	0.0028	0.0529	0.0293	0.000984	0.0314	0.0152
Sigmoid	0.0188	0.1370	0.0639	0.0155	0.1247	0.0603
Poly	0.0172	0.1310	0.0640	0.0146	0.1209	0.0619
Linear	0.0192	0.1386	0.0694	0.0169	0.1300	0.0681

**Table 6:** Neural network simulation output results and expected output results.

Algorithm Model	Simulation Output			Expected Output			Results
The GA-BP neural network	0.62745	0.00138	0.37117	1	0	0	Uncertainty
	0.68278	0.01471	0.30251	1	0	0	Uncertainty
	0.9969	0.0025	0.0006	1	0	0	Fire
	0.5986	0.3927	0.0087	1	0	0	Uncertainty
	0.99792	0.002	0.00008	1	0	0	Fire
The PSO-LSSVM network	0.86354	0.07689	0.07941	1	0	0	Fire
	0.87026	0.04597	0.0794	1	0	0	Fire
	0.64114	0.29953	0.05933	1	0	0	Uncertainty
	0.87451	0.11074	0.07941	1	0	0	Fire
	0.97166	0.00114	0.02283	1	0	0	Fire

**Table 7:** The results of the D-S evidence theory fusion approach.

Sample	*m*(*A*_1_)	*m*(*A*_2_)	*m*(*A*_3_)	Results
1	0.9997	0.0001	0.0002	Fire
2	0.9983	0.0011	0.0006	Fire
3	1	0	0	Fire
4	1	0	0	Fire
5	1	0	0	Fire

**Table 8:** A comparison of the real situation and system recognition.

Fire Types	Experiments/n	Alarms/n	Missed Alarms/n	Accuracy/%
Polyurethane fire	50	49	1	98%
Alcohol fire	50	48	2	96%
Beech wood smolder	50	48	2	96%
Cotton rope smolder	50	49	1	98%

**Table 9:** The GA-BP algorithm fusion results.

	Temperature/°C	Smoke/10^3^ ppm	CO/ppm	Fire Output	Smolder Output	No Fire Output
Node 1	25.6	2.36	6	0.6949	0.0532	0.2519
	31.6	3.68	26	0.7247	0.0513	0.2239
	35.9	2.56	17	0.9296	0.0011	0.0692
Node 2	26.3	1.84	2	0.6762	0.1522	0.1716
	33.7	2.28	14	0.8124	0.0524	0.1350
	36.9	2.44	7	0.9456	0.0325	0.0219
Node 3	25.3	2.6	6	0.7025	0.2305	0.0670
	32.4	1.95	23	0.7952	0.1248	0.0800
	37	1.6	9	0.9033	0.0362	0.0605

**Table 10:** The PSO-LSSVM algorithm fusion results.

	Temperature/°C	Smoke/10^3^ ppm	CO/ppm	Fire Output	Smolder Output	No Fire Output
Node 1	25.6	2.36	6	0.6314	0.1250	0.2436
	31.6	3.68	26	0.7615	0.0215	0.2171
	35.9	2.56	17	0.8703	0.0460	0.0838
Node 2	26.3	1.84	2	0.7043	0.1453	0.1504
	33.7	2.28	14	0.8021	0.1320	0.0659
	36.9	2.44	7	0.9382	0.0044	0.0573
Node 3	25.3	2.6	6	0.6125	0.2310	0.1566
	32.4	1.95	23	0.7493	0.0816	0.1691
	37	1.6	9	0.8853	0.0333	0.0814

**Table 11:** The D-S evidence theory fusion results.

	Temperature/°C	Smoke/10^3^ ppm	CO/ppm	Fire Output	Smolder Output	No Fire Output
Node 1	25.6	2.36	6	0.8658	0.0131	0.1211
	31.6	3.68	26	0.9174	0.0018	0.0808
	35.9	2.56	17	0.9928	0.0001	0.0071
Node 2	26.3	1.84	2	0.9086	0.0422	0.0492
	33.7	2.28	14	0.9763	0.0104	0.0133
	36.9	2.44	7	0.9984	0.0002	0.0014
Node 3	25.3	2.6	6	0.8710	0.1078	0.0212
	32.4	1.95	23	0.9617	0.0164	0.0218
	37	1.6	9	0.9924	0.0015	0.0061

## Data Availability

Not applicable.
